# Longitudinal profiling of antigen receptor gene repertoire dynamics in kidney transplant recipients after multiple SARS-CoV-2 vaccinations

**DOI:** 10.1093/immhor/vlag004

**Published:** 2026-02-20

**Authors:** Antonios Mingos, Nikolaos Pechlivanis, Georgios Karakatsoulis, Anastasia Anastasiadou, Glykeria Gkoliou, Nikolaos Vastarouchas, Alexandra Siorenta, Smaragdi Marinaki, Paraskevi Tsoutsoura, Myrto Papamentzelopoulou, Vassiliki Pitiriga, Mina Psichogiou, Angelos Hatzakis, Kostas Stamatopoulos, Elisavet Vlachonikola, Anastasia Chatzidimitriou

**Affiliations:** Institute of Applied Biosciences, Centre for Research and Technology Hellas, Thessaloniki, Greece; Department of Molecular Biology and Genetics, Democritus University of Thrace, Alexandroupoli, Greece; Institute of Applied Biosciences, Centre for Research and Technology Hellas, Thessaloniki, Greece; Institute of Applied Biosciences, Centre for Research and Technology Hellas, Thessaloniki, Greece; Institute of Applied Biosciences, Centre for Research and Technology Hellas, Thessaloniki, Greece; Institute of Applied Biosciences, Centre for Research and Technology Hellas, Thessaloniki, Greece; Institute of Applied Biosciences, Centre for Research and Technology Hellas, Thessaloniki, Greece; Immunology Department and National Tissue Typing Center, General Hospital of Athens “G. Gennimatas”, Athens, Greece; Nephrology Department and Renal Transplantation Unit, Laiko General Hospital, Medical School, National and Kapodistrian University of Athens, Athens, Greece; Nephrology Department and Renal Transplantation Unit, Laiko General Hospital, Medical School, National and Kapodistrian University of Athens, Athens, Greece; Molecular Biology Unit, 1st Department of Obstetrics and Gynecology, National and Kapodistrian University of Athens, Athens, Greece; Department of Microbiology, Medical School, National and Kapodistrian University of Athens, Athens, Greece; First Department of Internal Medicine, Laiko General Hospital, National and Kapodistrian University of Athens, Athens, Greece; Department of Hygiene, Epidemiology and Medical Statistics, Medical School, National and Kapodistrian University of Athens, Athens, Greece; Institute of Applied Biosciences, Centre for Research and Technology Hellas, Thessaloniki, Greece; Department of Molecular Medicine and Surgery, Karolinska Institute, Stockholm, Sweden; Institute of Applied Biosciences, Centre for Research and Technology Hellas, Thessaloniki, Greece; Institute of Applied Biosciences, Centre for Research and Technology Hellas, Thessaloniki, Greece; Department of Molecular Medicine and Surgery, Karolinska Institute, Stockholm, Sweden

**Keywords:** Antibodies, B Cells, T Cells, T Cell Receptors, Vaccination

## Abstract

Kidney transplant recipients (KTRs) exhibit impaired immune responses to vaccination against the severe acute respiratory syndrome coronavirus 2 (SARS-CoV-2) virus, remaining vulnerable to severe coronavirus disease 2019 (COVID-19) even after multiple vaccine doses. We hypothesized that repeated SARS-CoV-2 vaccinations in KTRs might promote remodeling of the adaptive immune repertoire. In order to address this hypothesis and gain insight into adaptive immune dynamics in this population, we employed next-generation sequencing (NGS) to determine longitudinal alterations in immunoglobulin (IG) and T cell receptor (TR) gene repertoires following multiple mRNA vaccinations and functional experiments to assess lymphocyte signaling capacity. TR gene repertoire analysis revealed increased diversity and reduced clonality after booster immunizations, indicative of substantial repertoire renewal. Although the relative frequency of SARS-CoV-2-specific TR clonotypes remained stable over time, significant shifts in TRBV gene usage reflected dynamic reshaping of the TR clonal architecture. Parallel IG gene repertoire profiling demonstrated increased diversity and limited oligoclonal expansions after booster mRNA vaccination. These changes were accompanied by elevated levels of somatic hypermutation in IG clonotypes similar to published SARS-CoV-2-specific clonotypes, suggestive of more efficient humoral responses following repeated antigenic exposure. Phospho-specific flow cytometry analysis revealed initially diminished B cell receptor signaling, which was restored following multiple immunizations, consistent with reversal of B cell anergy status. Altogether, our findings support the notion that repeated SARS-CoV-2 vaccinations drive the remodeling of cellular and humoral immune landscapes in KTRs. These results underscore the importance of tailored vaccination strategies to optimize immune protection in immunocompromised individuals.

## Introduction

Since the outbreak of severe acute respiratory syndrome coronavirus 2 (SARS-CoV-2) that led to the pandemic of coronavirus disease 2019 (COVID-19), vulnerable populations, including kidney transplant recipients (KTRs), have attracted research interest due to their high susceptibility to viral infections. Early findings demonstrated that KTRs face a significantly higher risk of severe COVID-19 outcomes, stemming from both their compromised immune systems and immunosuppressive treatments.^1^ To mitigate this risk, messenger RNA (mRNA) COVID-19 vaccines were rapidly administered to KTRs to elicit adequate immunity with minimal adverse effects.^2^^,^^3^

Initial observational studies revealed that KTRs showed suboptimal humoral responses following the standard 2-dose COVID-19 vaccination regimen, regardless of vaccine type, necessitating additional booster doses.^4–6^ That said, even with this approach, low seroconversion rates persisted among KTRs, suggesting diminished immune function.^7–10^ These findings underscore the need for a deeper characterization of both humoral and cellular immune responses to guide the development of innovative and effective prophylactic strategies tailored specifically for KTRs.^11^

Specific antigen recognition in the adaptive immune system is mediated by a remarkably diverse repertoire of antigen receptors: immunoglobulin (IG) on B cells and T cell receptor (TR) on T cells.^12–14^ Hence, IG/TR gene rearrangements encoding for IG/TR receptors are unique genetic markers that can be viewed as molecular signatures, instrumental for understanding immune responses,^15^ also in the context of infection by and/or vaccination against SARS-CoV-2.^16–19^

In this study, we hypothesized that repeated SARS-CoV-2 vaccinations in KTRs would drive the remodeling of the adaptive immune system, leading to renewal of the TR and IG gene repertoires, increased repertoire diversity and somatic hypermutation, as well as restoration of B cell receptor signaling capacity. To address this hypothesis, we characterized the TR and IG gene repertoires using targeted next generation sequencing (NGS), complemented by *in-silico* antigen specificity predictions. Particularly for B cells, we also assessed activation status using phospho-specific flow cytometry in order to determine the phosphorylation levels of key downstream molecules in the BcR signaling pathway.

## Materials and methods

### Study group

The study cohort included 38 individuals: (i) 19 kidney transplant recipients (KTRs) monitored at the Clinic of Nephrology and Kidney Transplantation of Laiko General Hospital, Athens, Greece; and (ii) 19 healthy individuals (HIs) as controls. HIs were healthcare workers at the Onassis Cardiac Surgery Center, Athens, Greece and the Laiko General Hospital, Athens, Greece. All KTRs were clinically stable throughout the study period, without rejection episodes or major treatment alterations (Table S1). Peripheral blood samples were collected from all participants under informed consent, in compliance with the Declaration of Helsinki. The study was approved by the Institutional Review Board of the participating institutions.

Sampling in KTRs was performed at 2 time points: 3 to 5 mo after either the completion of the primary two-dose vaccination series (T1) or after multiple vaccinations (T2: after the fourth or fifth dose). HIs were sampled once, either after receiving the standard two-dose vaccination or following documented SARS-CoV-2 infection.

Anti-RBD IgG levels were determined in all study participants by the Abbott SARS-CoV-2 IgG assay (Abbott Diagnostics), following the manufacturer’s instructions. Specifically, anti-RBD IgG levels in both KTRs and HIs were assessed at 7 to 14 d and again at 90 to 120 d post-two-dose vaccination or documented SARS-CoV-2 infection (specifically for the HIs). For KTRs, additional measurements were performed 30 d after the first repeated dose of vaccination (Table S2). All HIs developed adequate humoral immunity at least once, exhibiting a seroconversion threshold at 1,300 AU/ml of anti-RBD IgG antibodies in the serum. In contrast, none of the KTRs achieved sufficient humoral immune response levels at T1.

### Next generation sequencing: target amplification and library preparation

Peripheral blood mononuclear cells (PBMCs) were isolated from fresh blood samples by Ficoll centrifugation. Approximately 12 × 10^6^ cells per sample were used for downstream DNA/RNA extraction, following the AllPrep DNA/RNA/miRNA Universal Kit protocol (Qiagen, Hilden, Germany).

IG heavy chain variable domain gene rearrangements (IGHV-IGHD-IGHJ) were amplified by multiplex PCR with primers targeting conserved IGHV leader sequences and a consensus IGHJ primer for the different IGHJ genes.^20^ TR beta chain variable domain gene rearrangements (TRBV-TRBD-TRBJ) were amplified using the TCRB Gene Clonality Assay Tube A (Invivoscribe Technologies, San Diego, California, USA), as previously described.^21^

Library construction and next-generation sequencing (NGS) were conducted according to the manufacturer’s instructions (NEBNext Ultra II DNA library prep kit for Illumina, NEB, Ipswich, Massachusetts, USA), using the MiSeq reagent kit v3 (2 × 300 bp) on the MiSeq benchtop sequencer (Illumina, San Diego, California, USA). Each amplicon was sequenced using a paired-end protocol, providing double coverage of the complementarity determining region-3 (CDR3) of the TR and IG gene rearrangements.

### Next generation sequencing: bioinformatics analysis and interpretation

Quality assessment and strict filtering of raw NGS reads were performed, as previously described,^22^ prior to the stitching process, during which paired-end reads were combined into full-length sequences based on overlapping regions that captured the complete V-D-J rearrangement. Full-length sequencing reads were annotated using the IMGT/High-VQuest tool for the identification of V, D, and J genes and their junctions, and the calculation of somatic hypermutation (SHM) occurrences and germline identity in rearranged immunoglobulin heavy variable (IGHV) genes.^23^ Meta-data analysis was carried out using the T cell Receptor/Immunoglobulin Profiler (tripr) tool.^24^

For the meta-analysis, the term “clonotype” as utilized here corresponds to a productive IGHV-IGHD-IGHJ or TRBV-TRBD-TRBJ gene rearrangement defined by a particular pair of a specific V gene and a distinct CDR3 amino acid (aa) sequence. For TR gene rearrangements we set a 95% germline identity cutoff to exclude sequences with a high error rate. For IG gene rearrangements, lower values of germline identity were considered acceptable in order to assess SHM events.

The relative frequency of each clonotype/sample was calculated as the number of IGHV-IGHD-IGHJ or TRBV-TRBD-TRBJ gene rearrangements corresponding to this particular clonotype divided by the total number of productive, filtered-in IGHV-IGHD-IGHJ or TRBV-TRBDTRBJ gene rearrangements of that sample, respectively. The 10 clonotypes with the highest frequency within a given sample are herein referred to as “major.” In order to identify the fraction of clonal expansions that was represented at a meaningful frequency, we further defined “expanded clonotypes” based on a data-driven frequency distribution (2-sigma), performed for each sample within a group. The proposed cut-off threshold was defined as the median value of all individual thresholds, set at 0.05% for IG clonotypes and 0.33% for TR clonotypes, respectively.

Repertoire overlap was measured using the Jaccard Similarity Index (JI), calculated as the number of shared clonotypes between two samples or groups divided by the total number of clonotypes identified in both samples or groups. IGHV and TRBV gene frequencies were measured as the number of clonotypes expressing a specific V gene divided by the total clonotypes identified in a sample.

IG/TR gene repertoire diversity was estimated using the Recon algorithm, that calculates the observed species richness and entropy expressed as Hill numbers, while mathematical modeling in the background mitigates sampling biases, experimental errors, and possible variability in cell counts among samples.^25^^,^^26^ In detail, Hill numbers were calculated using q = 1, which corresponds to the Shannon diversity index and accounts for the abundance of each clonotype.

Repertoire clonality was estimated as: (i) the cumulative frequency of the expanded clonotypes per sample (CF-ex); and (ii) the cumulative frequency of major (top-10) clonotypes per sample (CF-10). Median values were used for the comparisons between groups and timepoints.

IG clonotypes were classified based on the IGHV germline identity of each clonotype (provided by the IMGT/High-VQuest tool) into three categories: (i) unmutated (UM, 100% germline identity), (ii) minimally mutated (MM, 99-99.9% germline identity), and (iii) mutated (M, <99% germline identity).

### In silico estimation of the specificity of IG and TR clonotypes against SARS-CoV-2

The specificity of TR clonotypes detected in KTRs against SARS-CoV-2-derived peptides was estimated in silico using the ERGO II binding prediction tool.^27^ TRB clonotypes, patients’ HLA allele information, and a data set of publicly available SARS-CoV-2 peptides from VDJdb were utilized as input for the prediction algorithm.^28^ In order to eliminate false positive results, an initial filtering step was applied to the HLA allele information by comparing each participant’s HLA genes with the experimentally validated HLA alleles known to present each peptide, as recorded in the VDJdb database. As a result, 4/19 KTRs and 2/19 HIs were excluded from the final specificity prediction analysis. TRB clonotypes with affinity scores >0.9 for SARS-CoV-2-derived peptides were further considered as SARS-CoV-2-specific.

The specificity of IG clonotypes against SARS-CoV-2 was determined using MAFFT, a multiple sequence aligner for amino acid or nucleotide sequences.^29^ MAFFT was used for the alignment of the VH CDR3 amino acid sequences against SARS-CoV-2-specific antibody sequences found in CoVAbDab.^30^ Strict criteria were applied to classify a particular clonotype as SARS-CoV-2-similar, including identical length of CDR3 amino acid sequence, at least 90% amino acid similarity, and no more than three amino acid differences between the aligned CDR3 sequences, minimizing false results.

TRB/IG clonotypes that counted for a single read (singletons) were excluded from the analysis.

### Cell cultures and in vitro stimulation experiments

PBMCs from 12 individuals (KTR-T1, *n* = 4; KTR-T2, *n* = 4; HIs, *n* = 4) were thawed and rested in culture medium (RPMI supplemented with L-glutamine, 10% heat-inactivated fetal bovine serum, 50 μg/ml penicillin/streptomycin, and 15 μg/ml gentamicin) at 37 °C in a humidified 5% CO_2_ atmosphere for approximately 1 h before proceeding with stimulation experiments. PBMCs were cultured at a density of 3 × 10^6^ cells/ml in the presence of different stimulants at distinct timepoints, as described below: (i) IG crosslinking: 10 μg/mL AffiniPure™ F(ab’)_2_ Fragment Goat Anti-Human IgM (Jackson ImmunoResearch Laboratories Inc., West Grove, Pennsylvania, USA), 20 μg/mL AffiniPure™ F(ab’)_2_ Fragment Goat Anti-Human IgG (Jackson ImmunoResearch Laboratories Inc., West Grove, Pennsylvania, USA); (ii) CD40L/IL-4 stimulation: soluble 0.1 μg/ml CD40L plus 1 μg/ml enhancer (Enzo Life Sciences, Farmingdale, New York, USA) in the presence of 3 ng/ml recombinant human IL-4 protein (R&D Systems, Minneapolis, Minnesota, USA).

### Flow cytometry

BcR signaling pathway activation after PBMC stimulation was assessed by flow cytometry. Data acquisition was performed using the Cytek Northern Lights instrument (Cytek Biosciences, Fremont, California, USA), and results were analyzed with the SpectroFlo software (Cytek Biosciences, Fremont, California, USA). The surface staining strategy was identical for both stimulation experiments, including CD45 (Anti-Hu CD45 Pacific Blue™, Exbio Antibodies, Prague, Czech Republic) and CD19 (Anti-Hu CD19 PE™, Exbio Antibodies, Prague, Czech Republic) cell markers to define the B-cell population, and CD38 (Anti-Hu CD38 PE-Cy5™, Exbio Antibodies, Prague, Czech Republic) as a B-cell activation marker. Intracellular staining was performed using the Fixation/Permeabilization Solution Kit (BD Biosciences, San Jose, California, USA), following the phosphoflow protocol from BD Biosciences (San Jose, California, USA). BcR signaling activation was evaluated by measuring phosphorylation changes in Erk (BD Phosflow™ Alexa Fluor® 488 Mouse Anti-ERK1/2 pT202/pY204), PLCγ2 (BD Phosflow™ Alexa Fluor® 647 Mouse anti-PLC-γ2 pY759), and p38 (BD Phosflow™ PerCP-Cy™5.5 Mouse anti-p38 MAPK pT180/pY182) following anti-IgM/anti-IgG stimulation. For the CD40L/IL-4 stimulation experiment, we measured the phosphorylation levels of Erk (BD Phosflow™ Alexa Fluor® 488 Mouse Anti-ERK1/2 pT202/pY204), NF-κB (BD Phosflow™ PE-Cy™7 Mouse anti-NF-κB p65 pS529), and Syk (BD Phosflow™ PerCP-Cy™5.5 Mouse Anti-ZAP70 pY319/Syk Y352).

### Statistical analysis

Repertoire diversity and clonality comparisons were conducted using non-parametric statistical tests, specifically the Mann–Whitney *U* test and the Wilcoxon signed-rank test. To evaluate differences in BcR responsiveness after stimulation between groups, changes within each group before and after stimulation were analyzed using the Kruskal–Wallis test. A significance level of 5% (*P *< 0.05) was applied to determine statistical significance, and all tests were 2-tailed.

## Results

### Repeated SARS-CoV-2 vaccinations promote TR gene repertoire renewal in kidney transplant recipients

TR gene repertoire analysis by NGS resulted in 9,230,695 sequences (mean: 161,942 sequences/sample). Further filtering and meta-analysis led to a final dataset of 567,800 distinct TRB clonotypes (mean: 9,961 clonotypes/sample) in KTRs and HIs.

**Figure 1.**
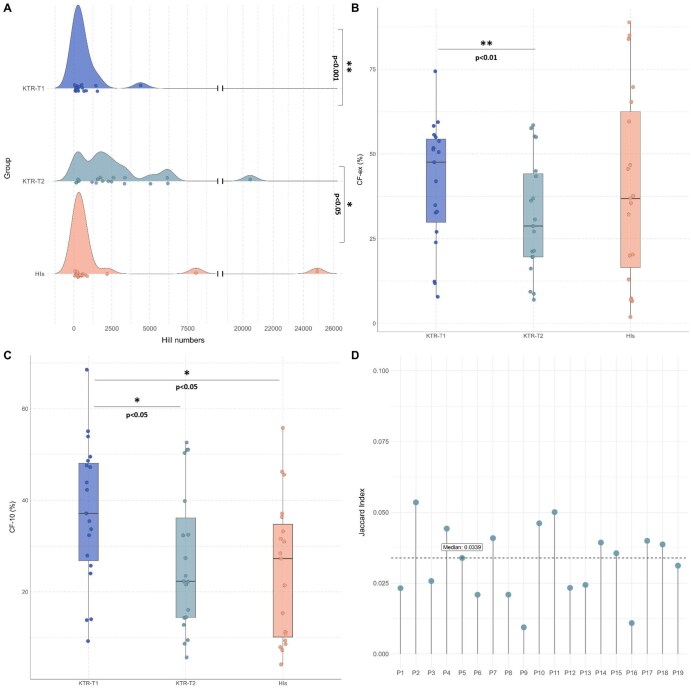
TR gene repertoire renewal following repeated SARS-CoV-2 vaccinations in KTRs. **(A)** Density ridge plots depict TR gene repertoire diversity across the three study groups: KTRs after the first (T1) and second (T2) vaccination timepoints, and healthy individuals (HIs). Diversity was measured using Hill numbers, with each dot representing an individual sample and the horizontal spread indicating distribution breadth. **(B–C)** Box plots represent the cumulative frequency (%) of expanded clonotypes (CF-ex) **(B)** and the cumulative frequency (%) of the major clonotypes (CF-10) **(C)** across the 3 examined groups. Each dot corresponds to an individual sample. **(D)** Bar plot shows the TR repertoire overlap between KTR-T1 and KTR-T2 overtime within individuals, measured using Jaccard similarity index. *P < 0.05, **P < 0.01, ***P < 0.001.

Multiple vaccinations led to a significant increase in TR repertoire diversity in KTR-T2 compared to either KTR-T1 (median Hill numbers: KTR-T1 = 297.9 vs KTR-T2 = 1,898, *P *< 0.001, Fig. 1A) or HIs (median Hill numbers: HIs = 337 vs KTR-T2 = 1,898, *P *< 0.05, Fig. 1A). Of note, the repertoire at T1 was primarily characterized by oligoclonal expansions, in contrast to T2 which displayed a significantly more polyclonal profile (median CFex: KTR-T1 = 52.5% vs KTR-T2 = 25.8%, *P *< 0.01, vs HIs = 46%, ns, Fig. 1B). Similar results were also obtained when only major clonotypes were taken into account (median CF-10: KTR-T1 = 37.1% vs KTR-T2 = 22.3%, *P *< 0.05, vs HIs = 27.3%, *P *< 0.05, Fig. 1C); however, the KTR-T1 group displayed a more oligoclonal TR profile compared to HIs. Notably, paired comparisons of the T1 and T2 repertoires in each case revealed only a minor fraction of shared clonotypes (median Jaccard Index = 0.03, range 0-1, Fig. 1D). This increase in repertoire diversity in KTRs following repeated vaccinations likely reflects the activation of a broader range of memory T lymphocytes, whereby initially smaller clones expand, allowing previously undetected TRB clonotypes to emerge, hence promoting overall repertoire diversity.

The TRBV gene repertoires of the KTR-T1 samples and HIs were similar, while a few significant differences were observed versus the KTR-T2 samples. In detail, the TRBV20-1 and TRBV28 genes were utilized at significantly (*P *< 0.05) higher frequencies in the KTR-T1 group (4.9% and 4.3%, respectively) and HIs (5.3% and 4.8%, respectively) compared to the KTR-T2 group (3.7% and 3.4%, respectively). Conversely, the TRBV7-2 gene was significantly (*P *< 0.05) more frequent in the KTR-T2 group (5.7%) than either the KTR1 group (2.7%) or HIs (2.5%).

### Overtime changes in the IG gene repertoire following multiple SARS-CoV-2 vaccinations in kidney transplant recipients

A total of 7,804,159 IG gene rearrangement sequences were obtained in all sample categories (mean: 136,915 sequences/sample). A total of 1,231,530 unique clonotypes (mean: 21,606 clonotypes/sample) were identified.

**Figure 2.**
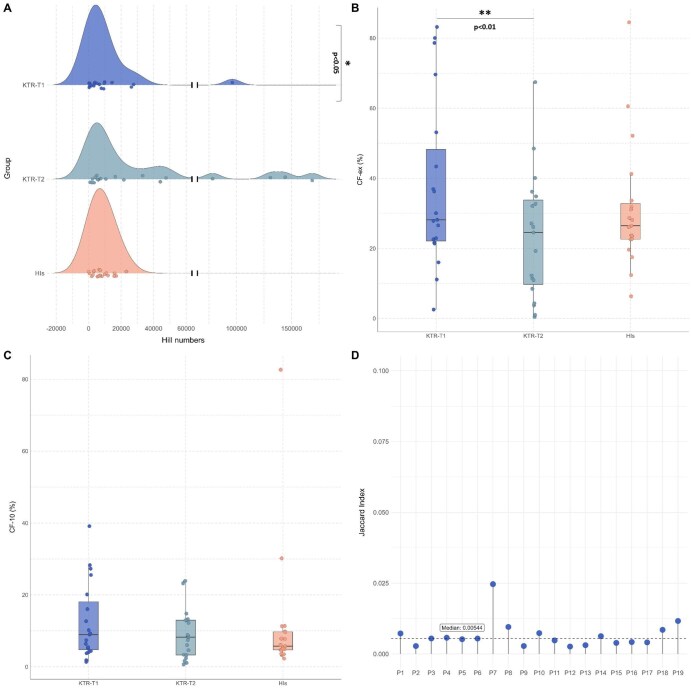
Repeated SARS-CoV-2 vaccination reshapes the IG gene repertoire in KTRs. **(A)** Distribution of IG gene repertoire diversity measured using Hill numbers, across KTRs, at the first and second vaccination timepoints, as well as HIs. **(B–C)** Box plots show the cumulative frequency of expanded clonotypes (CF-ex) **(B)** and the cumulative frequency of the major clonotypes (CF-10) **(C)**. Each dot represents an individual sample. **(D)** IG gene repertoire overlap between KTR-T1 and KTR-T2 groups, measured using the Jaccard similarity index for each individual. *P < 0.05, **P < 0.01, ***P < 0.001.

Similar to the TR gene repertoire, multiple vaccinations led to a significant increase of IG gene repertoire diversity in KTRs (median Hill numbers: KTR-T1 = 5,069 vs KTR-T2 = 10,571, *P *< 0.05, Fig. 2A), reflected in a more polyclonal profile at T2 (median CFex: KTR-T1 = 29% vs KTR-T2 = 24.5%, *P *< 0.01, Fig. 2B). However, no significant differences in diversity or clonality were identified between KTRs vs HIs (HI group: median Hill numbers = 6,987, median CFex = 26.5%, Fig. 2). A low Jaccard index value (median = 0.005, Fig. 2D) was identified when comparing T1 and T2 in KTRs, implying extensive IG gene repertoire renewal after multiple immunizations.

Distinct patterns in IGHV gene usage were identified across the three examined groups. High-frequent genes in both KTRs and HIs included IGHV4-34 (KTR-T1 = 7.3%, KTR-T2 = 6.9%, HIs = 9.3%) and IGHV4-59 (KTR-T1 = 6.2%, KTR-T2 = 5.5%, HIs = 7.4%), while the IGHV3-23 gene predominated in the KTR-T2 repertoire (KTR-T2 = 9%, KTR-T1 = 3.6%, HIs = 3.5%, *P *< 0.05).

### Predicted SARS-CoV-2 specificity of IG and TR clonotypes

In order to gain insight into the potential specificity of the detected IG and TR clonotypes we employed 2 metrics: (i) repertoire enrichment, defined as the number of clonotypes that were similar to published SARS-CoV-2-specific clonotypes relative to the total clonotypes; and, (ii) the cumulative frequency (CF %) of the predicted SARS-CoV-2-specific clonotypes.

**Figure 3.**
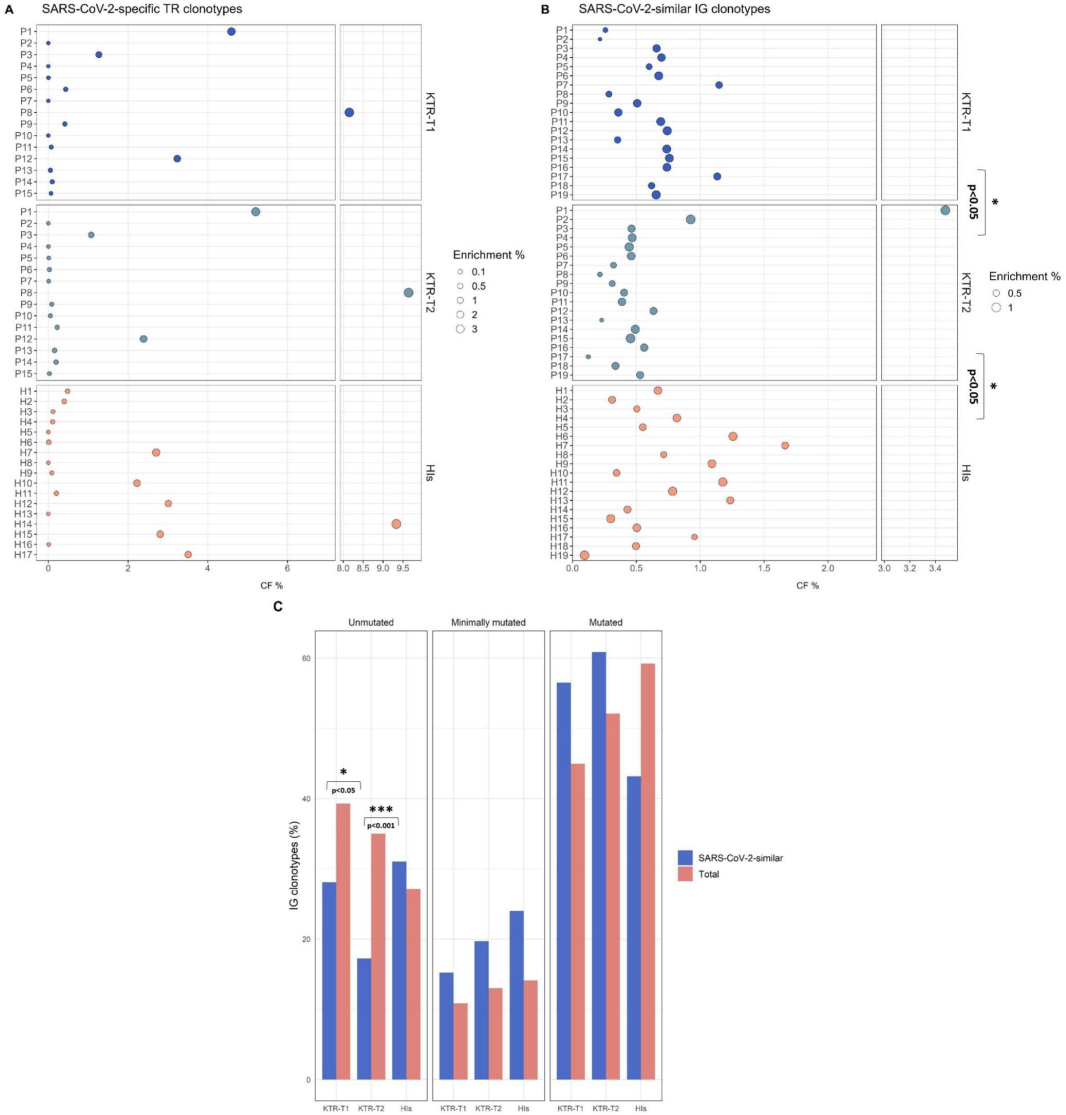
Prediction of SARS-CoV-2 reactivity of TR and IG clonotypes across KTRs and HIs, revealing distinct SHM profiles between groups. **(A–B)** Bubble plots present the cumulative frequency (CF%) of predicted SARS-CoV-2-specific TR clonotypes **(A)** and SARS-CoV-2-similar IG clonotypes **(B)** across KTRs and HIs. Each point represents the cumulative frequency of predicted clonotypes, calculated as the sum of the individual clonotype frequencies within each patient. Bubble size indicates the relative enrichment (%) of these clonotypes, corresponding to the proportion of clonotypes of interest relative to the total number of TR or IG clonotypes within the repertoire in that patient. **(C)** Bar plots depict the distribution of SARS-CoV-2-similar IG clonotypes (blue) enrichment (%) compared to the respective total IG gene repertoire (red) based on somatic hypermutation (SHM) status. IG clonotypes are categorized as unmutated (UM), minimally mutated (MM), and mutated (M). *P < 0.05, **P < 0.01, ***P < 0.001.

Following this approach, we identified a few TR clonotypes predicted as SARS-CoV-2-specific across both KTRs groups and HIs. Predicted SARS-CoV-2-specific clonotypes represented 0.11% and 0.10% of the total clonotypes in the KTR-T1 and KTR-T2 groups, respectively, with CF values of 0.08% and 0.12%, respectively (Fig. 3A). The group of HIs exhibited slightly higher percentages of predicted SARS-CoV-2-specific TR clonotypes, amounting to 0.21% of the total repertoire with a CF value of 0.38% (Fig. 3A).

**Figure 4.**
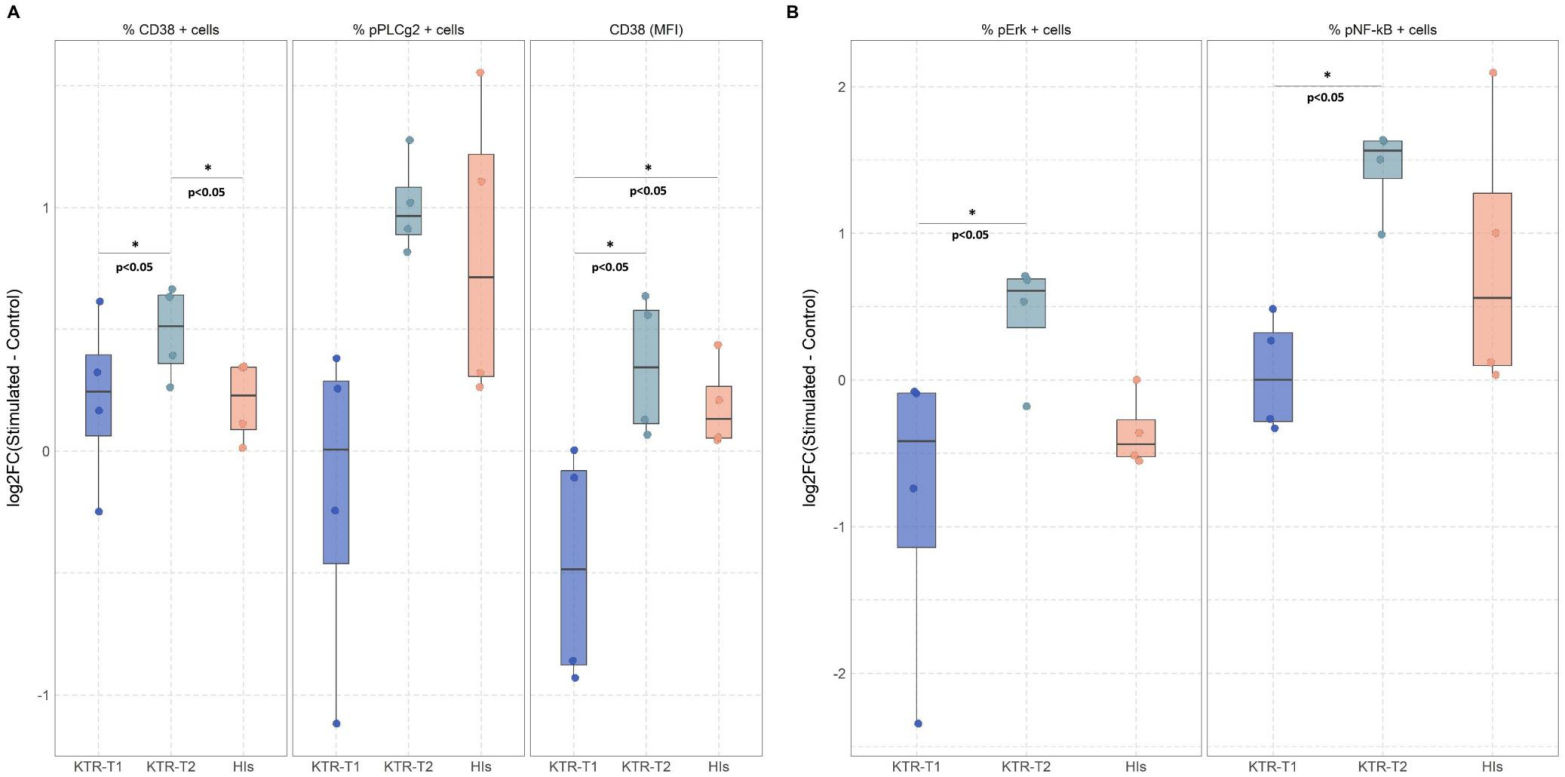
Restoration of BcR signaling capacity in KTRs following multiple SARS-CoV-2 vaccinations. BcR activation induced by stimulation was assessed by flow cytometry, calculated as the log2fold change (log2FC) between the stimulated and the control samples. **(A)** Activation induced by BcR crossliniking was evaluated by measuring log2FC in the percentage (%) of CD38^+^ cells, pPLCγ2^+^ cells, and the median fluorescence intensity (MFI) of CD38. **(B)** Activation induced by CD40L/IL-4 was estimated by measuring log2FC in the percentage (%) of the pErk^+^ and pNF-κB^+^ cells. Each dot represents the log2FC value for each individual. *P < 0.05, **P < 0.01, ***P < 0.001.

Predicted SARS-CoV-2-specific TR clonotypes in the three examined groups utilized different TRBV genes. Indicatively, the TRBV4-1 gene predominated in the KTR1 group (8.5%), thus differing significantly (*P *< 0.05) from either the KTR-T2 group (2.7%) or HIs (4%). In contrast, the TRBV12-3 gene was significantly (*P *< 0.05) more frequent in the KTR-T2 group (9.1%) and HIs (8.8%) compared to the KTR-T1 group (5.3%). Finally, the TRBV19 gene predominated in HIs (9.1%) compared to either the KTR-T1 (5.1%) or the KTR-T2 group (6.6%) (*P *< 0.05 for both comparisons).

Despite that anti-RBD IgG antibodies were undetectable in the KTR-T1 group, a minor fraction (0.72%) of IG clonotypes displayed high VH CDR3 sequence homology with SARS-CoV-2-specific clonotypes in public databases (Fig. 3B). The corresponding fraction in the IG gene repertoires of the KTR-T2 group and HIs were 0.65% and 0.63%, respectively (Fig. 3B). Nevertheless, SARS-CoV-2-similar IG clonotypes amounted to a significantly lower CF in KTR-T2 compared to KTR-T1 or HIs (median CF: KTR-T2 = 0.45% vs KTR-T1 = 0.66%, *P *< 0.05| vs HIs = 0.67%, *P *< 0.05, Fig. 3B).

Analysis of IGHV gene usage within the fraction of SARS-CoV-2-similar IG clonotypes revealed differences between the examined groups, albeit not reaching statistical significance, except for the IGHV3-23 gene which predominated in the KTR-T2 group (7.9%) compared to either the KTR-T1 group (2.6%) or HIs (2%) (*P *< 0.05 for both comparisons).

### The IG gene repertoire of SARS-CoV-2-similar clonotypes exhibits distinct somatic hypermutation patterns

We identified an overall similar load of somatic hypermutation (SHM) in all examined groups. Mutated IG clonotypes predominated (KTR-T1 = 45%, KTR-T2 = 52%, HIs = 59%, ns, Fig. 3C), whereas minimally mutated (MM) and unmutated (UM) clonotypes were less prevalent (MM: KTR-T1 = 11%, KTR-T2 = 13%, HIs = 14%, NS; UM: KTR-T1 = 39%, KTR-T1 = 35%, HIs = 27%, NS, Fig. 3C). Next, we analyzed SHM signatures in SARS-CoV-2-similar IG clonotypes and found that UM clonotypes were significantly more frequent in the KTR-T1 group (28.1%) and HIs (31%) compared to the KTR-T2 (17.2%) (*P *< 0.001 for both comparisons, Fig. 3C).

### Multiple vaccinations restore the dampened BcR signaling capacity in kidney transplant recipients

Using flow cytometry, we assessed the signaling capacity of B cells in KTRs compared to HIs, focusing on critical components of the BcR signaling pathway. BcR crosslinking revealed differences in BcR signaling across the 3 groups. In detail, the KTR-T2 group presented higher B-cell activation capacity compared to either the KTR-T1 group or HIs, estimated as the log2 fold change (log2FC) of the percentage (%) of CD38-positive cells between stimulated and control cells (log2FC: KTR-T1 = 0.24, KTR-T2 = 0.51, HIs = 0.23; *P *< 0.05, Fig. 4A). Moreover, significantly higher CD38 expression levels (MFI) were confirmed in both the KTR-T2 group and HIs compared to the KTR-T1 group (log2FC: KTR-T2 = 0.34 vs KTR-T1=-0.48, *P *< 0.05, HIs = 0.13 vs KTR-T1=-0.48, *P *< 0.05, Fig. 4A). PLCg2 phosphorylation levels were also found increased in the KTR-T2 group and HIs after stimulation, albeit without reaching statistical difference (log2FC: KTR-T1 = 0.01 vs KTR-T2 = 0.97, *P *= 0.1, KTR-T1 = 0.01 vs HIs = 0.71, *P *= 0.1, Fig. 4A).

CD40L/IL-4 stimulation experiments, designed to mimic T-cell-mediated BcR signaling activation, revealed differences in the BcR activation profile between the KTR-T1 and KTR-T2 groups. In detail, the KTR-T2 group demonstrated robust BcR signaling pathway activation after stimulation with CD40L/IL-4 for 45 min. Moreover, compared to the KTR-T1 group, it showed significantly (*P *< 0.05) increased phosphorylation of both Erk (log2FC values: 0.61 versus -0.42, respectively, Fig. 4B) and NF-κB (log2FC values: 1.57 vs 0.01, respectively, Fig. 4B). In contrast, the same treatment had no major effects in HIs (phospho-Erk: log2FC=-0.44, phospho-NF-κB: log2FC = 0.56, Fig. 4B).

## Discussion

A substantial proportion of KTRs fail to achieve sufficient humoral immunity to SARS-CoV-2 mRNA vaccination, remaining at risk of severe disease.^31^^,^^32^ While cellular immunity may partially counterbalance the deficit in humoral responses, there is insufficient evidence to suggest that cellular immunity alone can fully eliminate SARS-CoV-2, as also seen in infections by other coronaviruses.^33–36^ Indeed, most published studies primarily focused on humoral immunity with considerably less attention paid to cellular immune responses.^37^^,^^38^

In this study, we utilized NGS to longitudinally profile the IG and TR gene repertoire of KTRs, with healthy individuals (HIs) serving as a control group, with the aim to comprehensively understand how multiple vaccinations reshape the IG and TR clonal architecture in KTRs overtime.^39^^,^^40^ Flow cytometry experiments further assessed BcR signaling capacity.^41^^,^^42^ We found that multiple vaccination doses led to substantial TR repertoire reconstitution in KTRs, reflected in increased repertoire diversity at T2. Notably, relative to HIs, KTRs exhibited similar repertoire diversity at T1, whereas at T2 their TR repertoire was significantly more diverse. High TR repertoire diversity has previously been identified as a key factor in suppressing severe COVID-19, closely linked to increased SARS-CoV-2 neutralizing antibodies.^43^^,^^44^ Alterations in TRBV gene usage, along with minimal overlap of retained TR clonotypes between T1 and T2, underpin TR repertoire renewal.

Despite this, however, the relative fraction of predicted SARS-CoV-2-specific TR clonotypes remained unchanged between T1 and T2. That said, significant differences in TRBV gene usage were observed, consistent with published evidence for alterations in TR gene repertoire after booster vaccinations.^45^ Indeed, similar to studies in HIs,^45^ we identified shifts in clonal dominance and the emergence of new clonotypes over time in KTRs; however, differences in sampling time points between our study and the cited reference combined with different cohort sizes preclude further conclusions from being drawn. That notwithstanding, similar proportions of predicted SARS-CoV-2-specific TR clonotypes in both timepoints in KTRs may reflect sufficient cellular immunity. However, it remains uncertain whether cellular immunity alone can prevent severe COVID-19 in KTRs, rather than merely have a complementary role in providing long-term protection.^46^

Turning to the IG gene repertoire, we observed higher repertoire diversity following multiple vaccinations in KTRs, suggesting that repeated vaccination promotes activation of diverse memory clones overtime, although no major differences with HIs were detected. Differential IGHV gene usage between KTR-T1 and KTR-T2, at least for certain IGHV genes, further supports the notion of shifts in the IG repertoire, reflecting longitudinal immune response adaptations in KTRs. Focusing on the SARS-CoV-2-similar repertoire, we observed expanded IG clonotypes even at T1, with no major changes after multiple vaccinations.

Analysis of the SHM load revealed significantly higher proportions of unmutated or minimally mutated SARS-CoV-2-similar clonotypes in KTR-T1 and HIs compared to KTR-T2. This supports either an extrafollicular B cell response or, less plausibly, the presence of germinal center B cells with limited SHM in response to either vaccine- or virus-induced antigenic stimulation.^47^^,^^48^ Whatever the precise differentiation trajectory, our present findings are consistent with the results of studies conducted during the initial pandemic wave, which reported reactive SARS-CoV-2-specific B cells bearing low SHM levels, particularly in COVID-19 convalescents.^49–51^

Repeated vaccinations appeared to enhance SHM, as reflected in reduced proportions of UM SARS-CoV-2-similar clonotypes in KTR-T2, in keeping with other studies suggesting increased SHM after the third boost vaccination.^52^^,^^53^ Persistent germinal center reactions induced by multiple SARS-CoV-2 vaccinations, accompanied by increased IG repertoire diversity, have already been associated with high and broad neutralizing potency.^54–56^

BcR stimulation experiments confirmed dampened activation of the BcR signaling pathway in KTRs at T1, both through BcR crosslinking and CD40-mediated activation. This state of diminished BcR responsiveness at T1 fully aligns with the limited or absent IgG antibody production observed after two mRNA vaccine doses. Conversely, following multiple vaccinations, KTRs demonstrated robust responses to both BcR crosslinking and CD40L/IL-4 stimulation.^57^ Increased CD40L-mediated BcR responsiveness and TR repertoire renewal in KTRs at T2 may imply that T cells establish more robust interactions with B cells within germinal centers, contributing to more effective immune responses to antigenic stimulation, consistent with previously published studies.^58^ In keeping with this scenario, the phosphorylation status of NF-κB in B cells from KTRs was restored after repeated vaccinations to levels comparable with HIs.

Our study has certain limitations. First, we did not perform detailed phenotypic characterization of T and B cell subsets within each PBMC sample prior to sequencing. Hence, although all samples were processed using standardized protocols and equal input cell numbers were used, inter-sample variation in lymphocyte composition cannot be fully excluded. That said, to address potential biases during immunogenetic profiling, we estimated repertoire diversity using Hill numbers with q = 1, which accounts for clonotype frequencies within each sample rather than total cell counts. Second, the cohort size was relatively small, reflecting the challenges of obtaining longitudinal samples from KTRs in the era of pandemic. Particularly for the HIs, the lack of longitudinal samples represents another limitation, as it did not allow assessing changes in the immune repertoire. Finally, bulk TR beta chain sequencing, as performed in this study, does not allow obtaining information about the pairing of TR alpha and TR beta chains. That notwithstanding, while single-cell approaches would indeed provide a more comprehensive view of TR clonotypes, due to its greater combinatorial and junctional diversity, the TR-β chain is well established as the dominant determinant of antigen specificity,^27^^,^^59^ hence longitudinal TRB gene profiling offers meaningful insights into repertoire dynamics.

In conclusion, we highlight the dynamic evolution of TR and IG gene repertoires in KTRs following multiple SARS-CoV-2 vaccinations. TR repertoire renewal underlines the role of cellular immunity in supporting long-term immune protection. Concurrently, increased repertoire diversity and SHM levels in IG clonotypes suggest that repeated vaccinations lead to more efficient humoral responses consistent with restoration of B-cell signaling capacity revealed by signaling analysis. Together, these results reinforce the importance of booster vaccinations in optimizing immune responses in KTRs.

## Supplementary Material

vlag004_Supplementary_Data

## Data Availability

The next-generation sequencing data presented in the study are deposited in the European Nucleotide Archive under accession number PRJEB90224.
