# Supplementary material for: Longitudinal profiling of antigen receptor gene repertoire dynamics in kidney transplant recipients after multiple SARS-CoV-2 vaccinations
Source: Immunohorizons. 2026 Feb 20;10(2):vlag004. doi: 10.1093/immhor/vlag004 (PMC12925320; doi:10.1093/immhor/vlag004)
Supplement: vlag004_Supplementary_Data [file vlag004_supplementary_data.pdf]

Supplemental tables

Supplemental table 1. Clinical characteristics of kidney transplant recipients. This table summarizes, for each patient, the date of kidney transplantation, and the immunosuppressive regimens used at the time of transplantation. Abbreviations: MPA, mycophenolic acid; CSA, cyclosporine A.

Supplemental table 2. Longitudinal anti-RBD IgG measurements in study participants. Anti-RBD IgG antibody levels (AU/mL) are reported for each participant at three key time points: 7–14 days after completion of the primary 2-dose SARS-CoV-2 vaccination, 3–5 months post-primary vaccination or following SARS-CoV-2 infection, and 30 days after the third (booster) vaccination dose.

Supplemental table 1.

Patient ID	Transplantation date	Immunosuppressive regimens
1619	2003	NEORAL/MPA/CSA
1620	2018	ADVAGRAF
1621	2018	ADVAGRAF/TACROLIMUS
1622	2019	TACROLIMUS/MPA/CORTISONE
1623	2014	ADVAGRAF
1626	1997	MPA/CORTISONE
1627	1992	CELLCEPT/NEORAL
1653	2016	TACROLIMUS/ MYFORTIC/CORTICOSTEROID
1654	2010	ADVAGRAF/CELLCEPT/CORTISONE
1655	2012	MYFORTIC/PROGRAF/CORTISONE
1656	2012	ADVAGRAF/CORTISONE
1657	2011	ADVAGRAF/CELLCEPT/CORTISONE
1681	2008	ADVAGRAF/MEDROL/MYFORTIC
1682	2000	CELLCEPT/PROGRAF
1683	2019	MYFORTIC/PROGRAF/CORTISONE
1685	2017	ADVAGRAF/MYFORTIC/CORTISONE
1704	2012	ADVAGRAF/CELLCEPT/MEDROL
1705	2002	ADVAGRAF/CELLCEPT/CORTISONE
1706	2005	CERTICAN/CELLCEPT/MEDROL

Supplemental Table 2.

Patient ID	Group	7-14 days post primary 2-dose vaccination	3-5 months post primary 2-dose vaccination/SARS-CoV-2 infection	30 days post 3rd booster vaccination dose
1619	KTRs	1.1	3.3	1.4
1620	KTRs	57.1	66.8	1201.2
1621	KTRs	10.1	16.9	447.1
1622	KTRs	105	250.2	8027.0
1623	KTRs	135.3	395.7	19329.6
1626	KTRs	2.1	0.3	-
1627	KTRs	26.2	77.9	14597.7
1653	KTRs	3.7	7.6	-
1654	KTRs	27.9	45.2	7948.8
1655	KTRs	0.0	0.6	116.9
1656	KTRs	0.0	886.1	854.9
1657	KTRs	0.0	0	0.1
1681	KTRs	0.0	2.7	3.9
1682	KTRs	11.3	340.3	791.2
1683	KTRs	26.9	117.6	26876.6
1685	KTRs	3.3	0.8	-
1704	KTRs	0	0.3	0.6
1705	KTRs	4.7	3.2	9.6
1706	KTRs	0	2.9	128.6
1586	HIs	12,970.5	791.3	-
1569	HIs	24,146.7	2,016.8	-
1567	HIs	5,205.2	405.0	-
1571	HIs	15,017.8	947.3	-
1572	HIs	6,361.7	628.9	-
1651	HIs	30,435.2	-	-
1600	HIs	5,592.8	375.6	-
1583	HIs	14,368.0	817.7	-
1570	HIs	24,010.9	2,850.4	-
1703	HIs	5,729.4	619.2	-
1585	HIs	11,640.6	748.1	-
1566	HIs	-	6,491.0	-
1568	HIs	-	2,799.6	-
1573	HIs	-	3,259.7	-
1580	HIs	-	1,834.1	-
1587	HIs	-	3,248.4	-
1629	HIs	-	1,383.9	-
1630	HIs	-	2,471.4	-
1658	HIs	-	2,094.4	-
